# Mechanistic study of a diazo dye degradation by Soybean Peroxidase

**DOI:** 10.1186/1752-153X-7-93

**Published:** 2013-05-27

**Authors:** Umme Kalsoom, Syed Salman Ashraf, Mohammed A Meetani, Muhammad A Rauf, Haq Nawaz Bhatti

**Affiliations:** 1 Department of Chemistry & Biochemistry, University of Agriculture, Faisalabad, Pakistan; 2 Department of Chemistry, UAE University, P. O. Box 15551, Al-Ain, United Arab Emirates

**Keywords:** Azo dye, Trypan Blue, Phenol dye, Enzymes, Degradation, Mechanism, Soybean peroxidase, LC-MS/MS

## Abstract

**Background:**

Enzyme based remediation of wastewater is emerging as a novel, efficient and environmentally-friendlier approach. However, studies showing detailed mechanisms of enzyme mediated degradation of organic pollutants are not widely published.

**Results:**

The present report describes a detailed study on the use of Soybean Peroxidase to efficiently degrade Trypan Blue, a diazo dye. In addition to examining various parameters that can affect the dye degradation ability of the enzyme, such as enzyme and H_2_O_2_ concentration, reaction pH and temperature, we carried out a detailed mechanistic study of Trypan Blue degradation. HPLC-DAD and LC-MS/MS studies were carried out to confirm dye degradation and analyze the intermediate metabolites and develop a detailed mechanistic dye degradation pathway.

**Conclusion:**

We report that Soybean peroxidase causes Trypan Blue degradation via symmetrical azo bond cleavage and subsequent radical-initiated ring opening of the metabolites. Interestingly, our results also show that no high molecular weight polymers were produced during the peroxidase-H_2_O_2_ mediated degradation of the phenolic Trypan Blue.

## Introduction

Textile dyes are aromatic compounds representing a major class of organic pollutants that are found in the waste effluent discharged by different industries such as textile, petroleum refining, paper and pulp, leather and plastics, wood preservation, etc. These compounds are toxic and potentially carcinogenic in nature and must be removed from industrial effluent before entering into the larger water bodies such as ponds, rivers and lakes [1-4]. Conventional methods of removing such pollutants such as adsorption, sedimentation, coagulation, and filtration result in a secondary waste which in itself is a problem to dispose [5-7]. Chemical methods, such as advanced oxidation techniques, have been adopted for handling such effluent [8], but have limited success so far at a commercial scale [9].

The process of removing the contaminants in the environment by biological methods using the metabolic potential of microorganisms to degrade a wide variety of pollutants has been explored with some success. The main advantage of bioremediation is its reduced cost compared to conventional techniques [10,11]. Furthermore, the method is environmentally friendlier as it leaves the local ecosystem intact and does not add any variables to it. The bioremediation process can also deal with lower concentrations of contaminants, whereby the cleanup by physical or chemical methods would not be feasible.

Although a number of microorganisms including bacteria, fungi, and yeasts have been found to decolorize textile dyes, biocatalysts such as enzymes have also been used for the degradation and mineralization of dyes [12,13]. Enzymes can specifically react with organic pollutants and remove them by transforming them into other products. The catalytic action of enzymes is generally very efficient and selective as compared to chemical catalysts due to their higher reaction rates, milder reaction conditions and greater stereo-specificity. They can catalyze reactions at relatively low temperature and in the entire aqueous phase pH range.

Since dye molecules display a high structural variety, they are degraded by only few enzymes. These biocatalysts have one common mechanistic feature, i.e., they are all redox-active molecules and thus exhibit relatively wide substrate specificities. The enzymes are responsible for generating highly reactive free radicals that undergo a complex series of spontaneous cleavage reactions. Some common examples of enzymes used for dye degradation are lignin peroxidase, laccases, horseradish peroxidase, tyrosinase, Manganese peroxidase etc. [14-16]. Peroxidases that have been used for treatment of aqueous aromatic contaminants and degradation of dyes include horseradish peroxidase (HRP), lignin peroxidase (LiP) and a number of other peroxidases from different sources. Although there are numerous reports on the use of peroxidases for the degradation of organo-pollutants, studies that show detailed mechanistic pathway of dye breakdown are very few.

In the present study, we report on the use of commercial soybean peroxidase (SBP) for the degradation of a di-azo dye, Trypan Blue. Effect of parameters such as aqueous phase pH, H_2_O_2_ and SBP concentration, contact time and application of immobilized SBP and dye concentration has been investigated to optimize the dye degradation. Furthermore, the performance of the dye removal process by SBP immobilized in polyacrylamide gel matrix was evaluated in order to study the enzyme’s reusability. Most importantly, we used LC/MS-MS to identify various intermediates and develop a detailed mechanism for the degradation of this di-azo compound by SBP. Such detailed mechanistic degradation schemes for peroxidase-mediated dye degradation are important to understand the peroxidase mechanism and are unfortunately not widely published.

## Experimental

**Figure 1:**
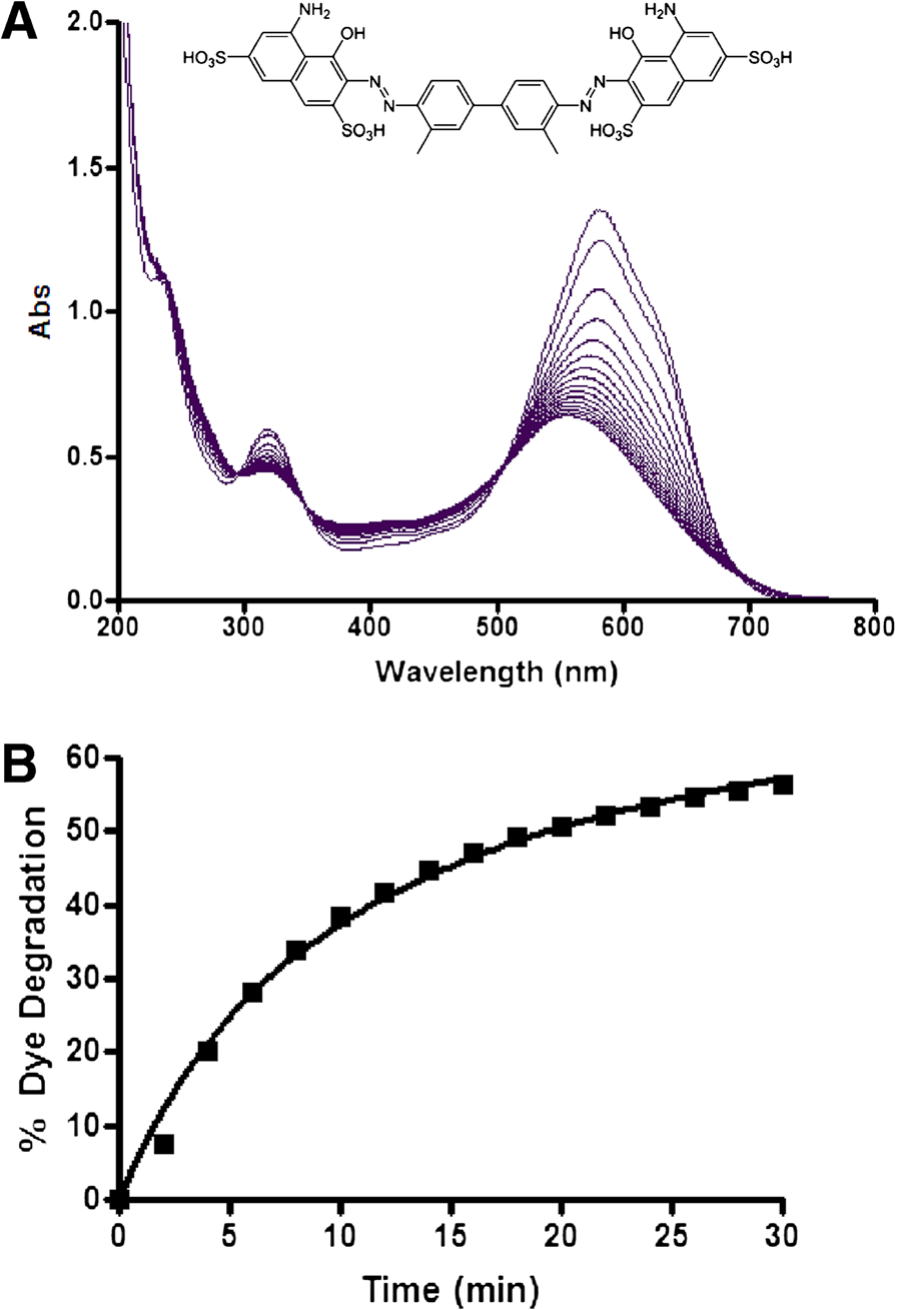
**SBP mediated degradation of Trypan Blue. A)** Trypan Blue (20 ppm) was incubated with 1 mM H_2_O_2_ with 10 U/ml SBP at pH 7 and UV-Visible spectra were recorded every 2 minutes (till 30 minutes). **B)** Trypan Blue % degradation (calculated from A_595_) as function of time.

Trypan Blue (Molecular Formula = C_34_H_28_N_6_O_14_S_4,_ FW = 872.88 g mol^−1^), herein abbreviated as TB, was procured from Clariant chemicals and used as such. Its molecular structure is shown in Figure 1. All the other chemicals used in this work were obtained from Aldrich and were of high purity ( > 98% ).

Soybean Peroxidase (SBP) used in this study was obtained from a commercial supplier (Bio-Research Products, Inc, USA) and was used as supplied. The specific activity of the enzyme was 2,700 U/mg and was supplied as a 26 μM solution, which was stored at 4°C.

Trypan Blue (TB) stock solution of 2,000 ppm was prepared in a 250 mL flask by first dissolving an appropriate amount of the dye in deionized water. Necessary dilutions of this stock were further done as per the requirement of the experiment. Unless otherwise indicated, the working concentration of the TB dye was 40 ppm. Dye degradation reactions were carried out by adding H_2_O_2_ to a buffered dye solution containing the SBP enzyme. Spectrophotometric measurements were made either using a CARY 50 UV/Vis spectrophotometer (3 ml reaction volume) or a Tecan Sunrise microplate reader (200 μl reaction volume).

For effect of temperature on dye degradation experiments, a temperature-controlled plate reader (Perkin Elmer, Victor X) set at appropriate temperature was used.

The absorbance value obtained in each case was plotted against time to obtain the order of % degradation. The % degradation for the dye was calculated by observing the changes in λ_max_ (595 nm) of the solution. The studies were carried out at 25°C otherwise indicated. For pH studies, the dye solution was prepared in 33.33 mM buffers adjusted to specific pH using 0.1M glycine/HCl, acetate, phosphate and Tris/HCl buffers. These buffers did not cause any change in the λ_max_ of the dye.

Polyacrylamide (PA) entrapped SBP was prepared as follows: 4.25 ml of potassium phosphate buffer (0.1M, pH = 7.0) was mixed with 2.7 ml of acrylamide solution (3g acrylamide and 0.08g of bisacrylamide in 10 ml potassium phosphate buffer) and 80 μl of ammonium persulfate solution (10% ammonium per sulfate in potassium phosphate buffer) and the resulting mixture was mixed in 20 ml vial. Subsequently, 0.45 ml of SBP solution (containing 1,215 units) was added followed by 10 μl of TEMED (N,N,N,N-tetra methylethylenediamine) reagent and the mixture was gently mixed. The complete polymerization of acrylamide/bisacrylamide took about 30 minutes (at room temperature). Gel was transferred subsequently to vacuum filter system to remove the solution and subsequently washed with phosphate buffer. Gel was broken by aspiration using a sharp knife into small equal size pieces (~ 0.5 cm x 0.5 cm x 0.5 cm) and stored at 4°C prior to use.

High performance liquid chromatography (HPLC) and LC/MS analyses were carried out as previously described [17]. Briefly, an Agilent HP 1100 liquid chromatography system, (Agilent, USA) with an Agilent Zorbax® SB-C18 column 150 mm x 4.6 mm packed with 5 μm particle size, coupled to a diode array detector (Agilent, USA) and an ion trap 6310 mass spectrometer (Agilent technologies) were used to monitor and indentify dye degradation metabolites. The mobile phase consisted of solution A (0.1 M ammonium formate (pH 6.7)) and solution B (1:1 acetonitrile/methanol) and a gradient from 0% B to 80% B in 40 minutes at the flow rate was 1 mL/min was used to obtain the chromatographs. The mass spectrometer was equipped with an electrospray ionization source and operated in positive polarity. The ESI conditions were as follows: capillary voltage: 3.5 kV, endplate offset was fixed at 500 V; skimmer at 40 V; trap drive at 53 V; the nebulizer pressure was 70 psi and drying temperature was 350°C. The mass range was from 50 to 1200 m/z. Tandem MS experiment was done using the Auto MSn mode wherein Helium gas was used as a collision gas.

## Results and discussion

### Degradation of the Trypan Blue solutions

The absorption spectrum of an aqueous solution of Trypan Blue (TB) in the range of 200–800 nm is shown in Figure 1. The spectrum was characterized by the presence of an intense band in the visible region at 595 nm, which gives the solution a blue color. As shown by others for different peroxidases [18,19], upon addition of Soybean Peroxidase (SBP) and H_2_O_2,_ the solution undergoes rapid degradation as shown in Figure 1A. The percentage change of degradation of the dye was calculated as follows:

(1) $$\%\;\mathrm{degradation}=\left[\left(A_{o}-A_{t}\right)/A_{o}\right]\times 100$$

where A_0_ is the initial absorbance of dye solution and A_t_ is the absorbance of the dye solution at any given time. The results are shown in Figure 1B. One can see from this figure that in the early stages, the % degradation was fast which became slow with time and almost leveled off around 30 minutes. The addition of SBP or H_2_O_2_ alone did not show any degradation of the dye.

### Effect of SBP on dye degradation

The removal of an organic pollutant is dependent on the amount of catalyst added and the contact time. There is thus an optimum relationship between the concentration of enzyme and substrate for achieving maximum activity. To study the optimum dose of SBP, experiments were carried out at various SBP doses ranging from 10 to 80 units/ml at specified experimental conditions (TB = 40 ppm, pH = 7; temperature = 25°C, contact time = 10 min, H_2_O_2_ = 64 μM). The results are shown in Table 1. The enzyme dose was found to have significant influence on dye removal reaction. The increase in the SBP dose from 10 units/ml to 80 units/ml resulted in a gradual increase in the dye removal (16– 64%) and seems to be leveling off at 80 units/ml. Table 1 also gives the % degradation of the dye solution with changing SBP amount. As seen for the rate of dye degradation, the % dye degradation also increased with increasing SBP concentration, but showed lesser increase in % degradation at higher enzyme concentrations. This can explained on the basis that in the initial stages, the reaction between dye and SBP is quite fast and becomes slower when SBP increases, as there is not enough dye molecules available for the reaction. Similar trend has also been reported with chloroperoxidase-mediated degradation of Sunset Yellow dye [19].

**Table 1:** The effect of SBP concentration on Trypan Blue degradation and the rate of dye degradation

	**% Dye Degradation**		**Initial Rate (min**^**-1**^**)**	
**[SBP], U/ml**	**Mean**	**SD**	**Mean**	**SD**
10	34.9	0.1	0.169	0.001
20	43.6	1.3	0.341	0.013
40	57.8	1.5	0.423	0.011
80	62.3	3.3	0.637	0.049

[Dye] = 40 ppm, [H_2_O_2_] = 64 μM, pH = 7 (10 minutes).

### Effect of dye concentration

The concentration of the substrate present in the reaction mixture directly effects enzyme-mediated reactions. The rate of the reaction should increase and reach the maximum value if the amount of enzyme concentration is kept constant and the substrate concentration is gradually increased. Once the saturation is achieved (maximum enzyme velocity, ν_max_), any further addition of the substrate will not change the rate of reaction. Studies in this regard were carried out at different concentrations of the dye (10, 20, 40 and 80 ppm), while keeping all the other parameters constant (H_2_O_2_ = 64 μM; pH = 7; temperature = 25°C). With the increase in dye concentration, the removal was found to be most effective at the lowest dye concentration (10 ppm). Further increase in dye concentration up to 80 ppm resulted in relatively slower dye removal (Additional file 1: Figures S1) – at 80 ppm (92 μM TB) the dye degradation observed of about 60% was consistent with the H_2_O_2_:TB ratio of 2:3. A similar profile for studying the oxidation of 2,4-chlorophenol oxidation by horseradish peroxidase has also been reported in the literature [20].

### Effect of H_2_O_2_ addition

**Figure 2:**
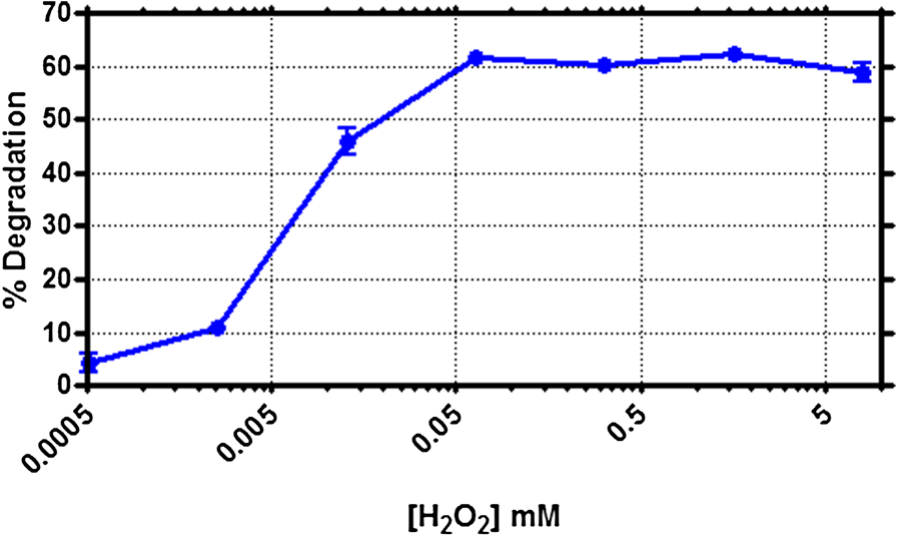
**Effect of hydrogen peroxide concentration on SBP mediated Trypan Blue degradation.** Trypan Blue (10 ppm) was incubated with 40 U/ml of SBP in the presence of increasing concentrations of H_2_O_2_ at pH 7 and the dye degradation was measured after 10 minutes of enzymatic reaction.

Hydrogen peroxide reacts with the peroxidase, to oxidize the native enzyme to form an enzyme intermediate, which accepts the aromatic compound to carry out its oxidation to a free radical form. In this regard, experiments were done wherein the % dye degradation was measured as a function of H_2_O_2_ concentration, while keeping the other parameters constant (TB = 10 ppm, pH = 7; temperature = 25°C). The results obtained are shown in Figure 2. As can be seen (and expected), increasing H_2_O_2_ concentration led to increased dye degradation. However, after reaching the maximum dye degradation with 64 μM H_2_O_2_, further increase in H_2_O_2_ did not cause any additional dye degradation. On the contrary higher H_2_O_2_ was detrimental to the process, most likely due to damaging the enzyme itself. This shows the critical significance of optimizing H_2_O_2_ concentrations in these enzyme-based dye degradation approaches [19]. Hydrogen peroxide concentrations were also optimized at higher dye concentrations (20 – 80 ppm) and the results showed that 64 μM was the optimum H_2_O_2_ concentration for 10–40 ppm but much higher H_2_O_2_ concentration was necessary for 80 ppm dye concentration (Additional file 2: figure S2, Additional file 3: Figure S3, and Additional file 4: Figure S4).

**Figure 3:**
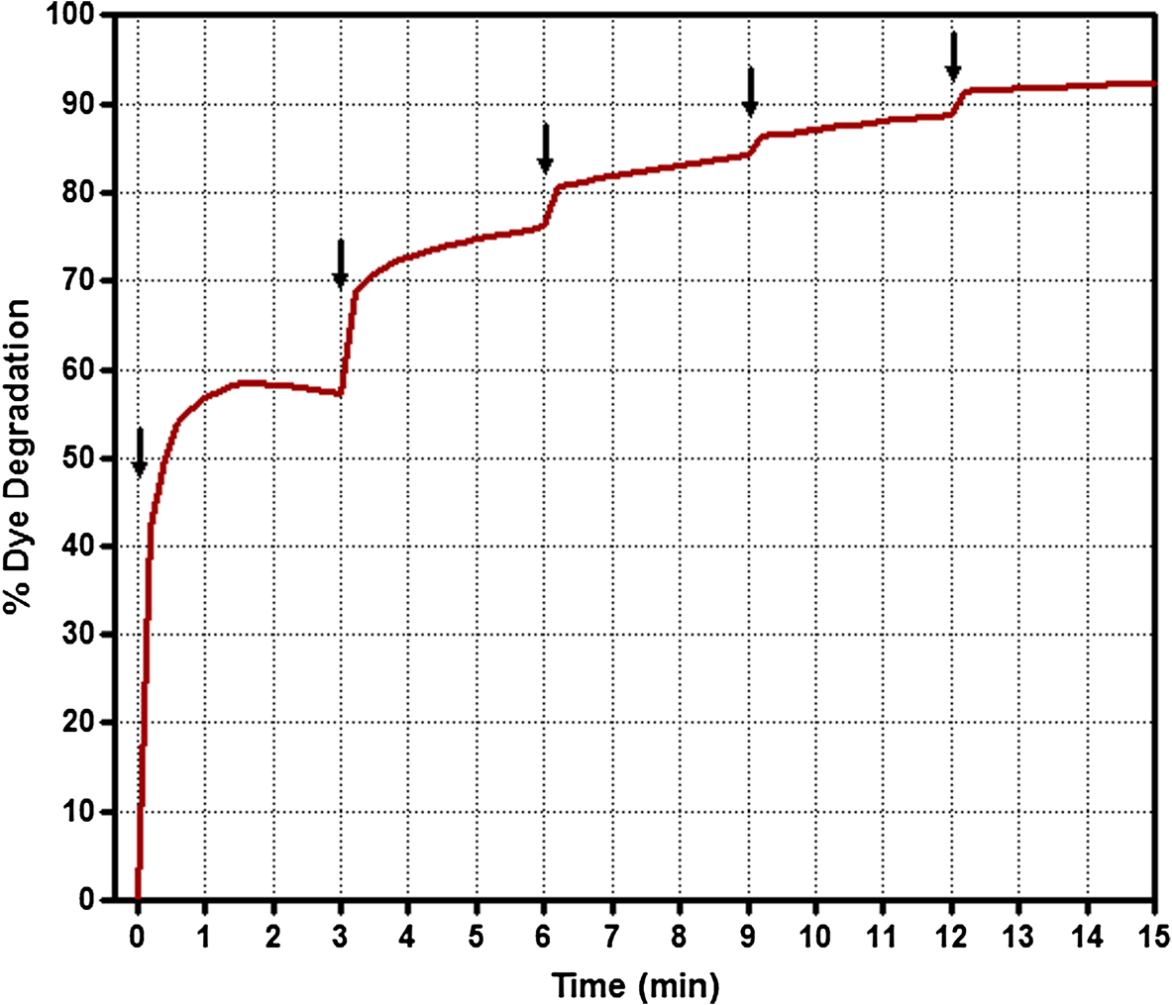
**Use of sequential addition of hydrogen peroxide to achieve almost complete degradation of Trypan Blue with SBP.** Trypan Blue (40 ppm) was incubated with 40 U/ml SBP in pH 4 and the dye degradation reaction was initiated with the addition of 64 μM H_2_O_2_. After the reaction seemed to have leveled off additional H_2_O_2_ (64 μM) were added as indicated by arrows.

In order to achieve complete dye degradation and to clearly understand the role of H_2_O_2_ in SBP-mediated Trypan Blue degradation, H_2_O_2_ was added in sequential increments to the dye reaction mixture and the resulting % dye remaining was noted. The results are shown in Figure 3. As one can see from this figure that when initial amount of H_2_O_2_ (64 μM) was added, the dye degradation immediately increased to about 60% in 3 minutes. At this stage all the H_2_O_2_ seems to have been consumed. The % degradation became more (75% in 6 minutes) after another addition of (64 μM) H_2_O_2_ while the added H_2_O_2_ is consumed in the process. A step wise continuous addition of H_2_O_2_ caused further degradation of the TB solution till more than 90% of the degradation was observed in less than 15 minutes.

### Effect of pH

**Figure 4:**
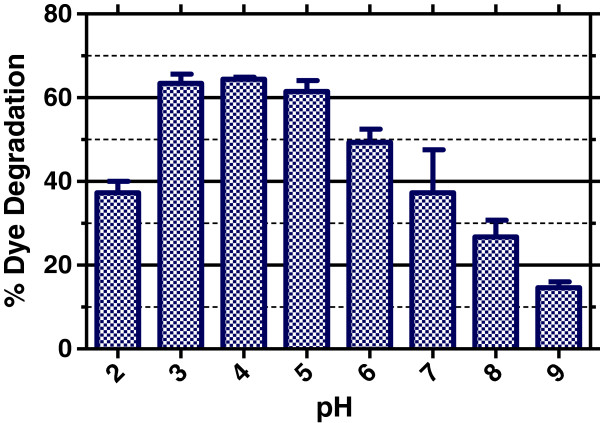
**Effect of pH on SBP mediated Trypan Blue degradation.** [Dye] = 40 ppm, [H_2_O_2_] = 64 μM, [SBP] = 40 U/ml, [buffer] = 33.33 mM. The data shows % dye degradation after 5 minutes (averages and standard deviation of quadruplicate measurements).

Dye degradation and enzymatic reactions are well known to be pH dependent. In order to optimize this parameter, SBP-mediated dye degradation was studied at different pH values (from 2 to 9), while keeping the other conditions same (TB = 40 ppm; buffer = 33.33 mM; H_2_O_2_ = 64 μM; temperature = 25°C). The results are shown in Figure 4. These experiments show that the degradation process is critically dependent on the pH of the solution: the enzyme activity increases dramatically by changing the pH from 2 to 7. At higher pH values of 8 and 9, the dye shows significantly poor degradation. The optimum pH for SBP-mediated Trypan Blue degradation appeared to be pH 4, where almost 60% of the dye could be degraded in one minute. Surprisingly, SBP was also very active at lower pH values of 2 and 3. At pH 3, SBP was almost comparable in activity as pH 4 (optimum pH), whereas even at pH 2, 40% of the dye could be degraded. This is similar to what others have reported [19,21] and underscores the usefulness of this enzyme to degrade industrial effluents which may be very acidic in nature. This pH dependence can be explained on the basis of the following mechanism which has been reported previously in the literature reviewed in [10].

The catalytic cycle of peroxidases involves the formation of two intermediates, Compound I and Compound II, according to the following reactions:

(2) $$\mathrm{Peroxidase}+H_{2}O_{2}\to \mathrm{Compound}\;I+H_{2}O$$

(3) CompoundI+SH→CompoundII+S•

(4) $$\mathrm{Compound}\;\mathrm{II}+\mathrm{SH}\to \mathrm{Peroxidase}+S•+H_{2}O$$

(SH indicates a generic substrate)

The above mentioned reactions steps of the catalytic cycle are pH dependent and work best under acidic conditions. In the first step (2), the formation of compound I is favored by the presence of a network of hydrogen bonds between the Fe-heme/H_2_O_2_ adduct and the distal histidine and arginine side chains, whereas, in the other steps (3) and (4), the substrate oxidation depends on its protonation state [20].

### Effect of temperature

**Figure 5:**
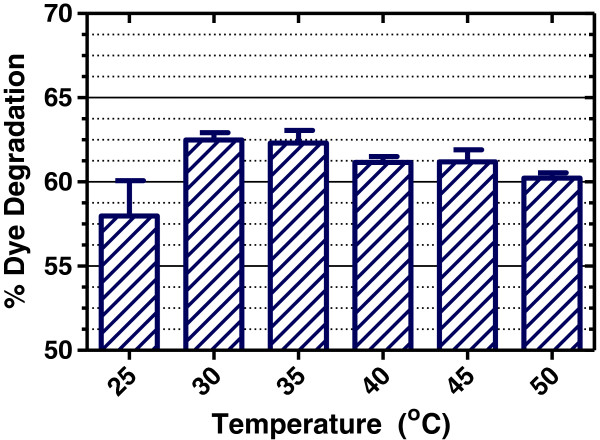
**Thermal stability of SBP in the range of 25 – 50°C.** [Dye] = 40 ppm, [H_2_O_2_] = 64 μM, [SBP] = 40 U/ml, pH 7. The data shows % dye degradation after 5 minutes (averages and standard deviation of quadruplicate measurements).

The dye degradation was also monitored as a function of temperature. SBP appears to work well at 25°C; however, increasing the temperature caused slightly higher degradation, as shown in Figure 5. Interestingly, SBP was found to be active even at high temperatures (50°C), this is unusual as normally protein and enzymes can be thermally denatured and deactivated at higher temperatures. In fact, SBP has been shown to be a thermally stable enzyme with T_m_ = 90.5°C at pH 8.0 buffer with 1 mM CaCl_2_[22]. In addition to high thermal stability and being able to tolerate highly acidic conditions, it has been reported that SBP is also very resistant to denaturation by guanidine hydrochloride [23]. These properties (high enzymatic activity in very acidic pH and high thermal and structural stability) points to additional industrial applications of this very interesting enzyme.

In summary, SBP was found to be very effective for the degradation of TB, and under optimal conditions could degrade the dye very quickly. Similar results were also observed for another reactive dye namely Turquoise Blue where SBP was used for the degradation resulting in 95% degradation of the dye [21].

### Degradation of dye with Polyacrylamide (PA)-entrapped SBP

**Figure 6:**
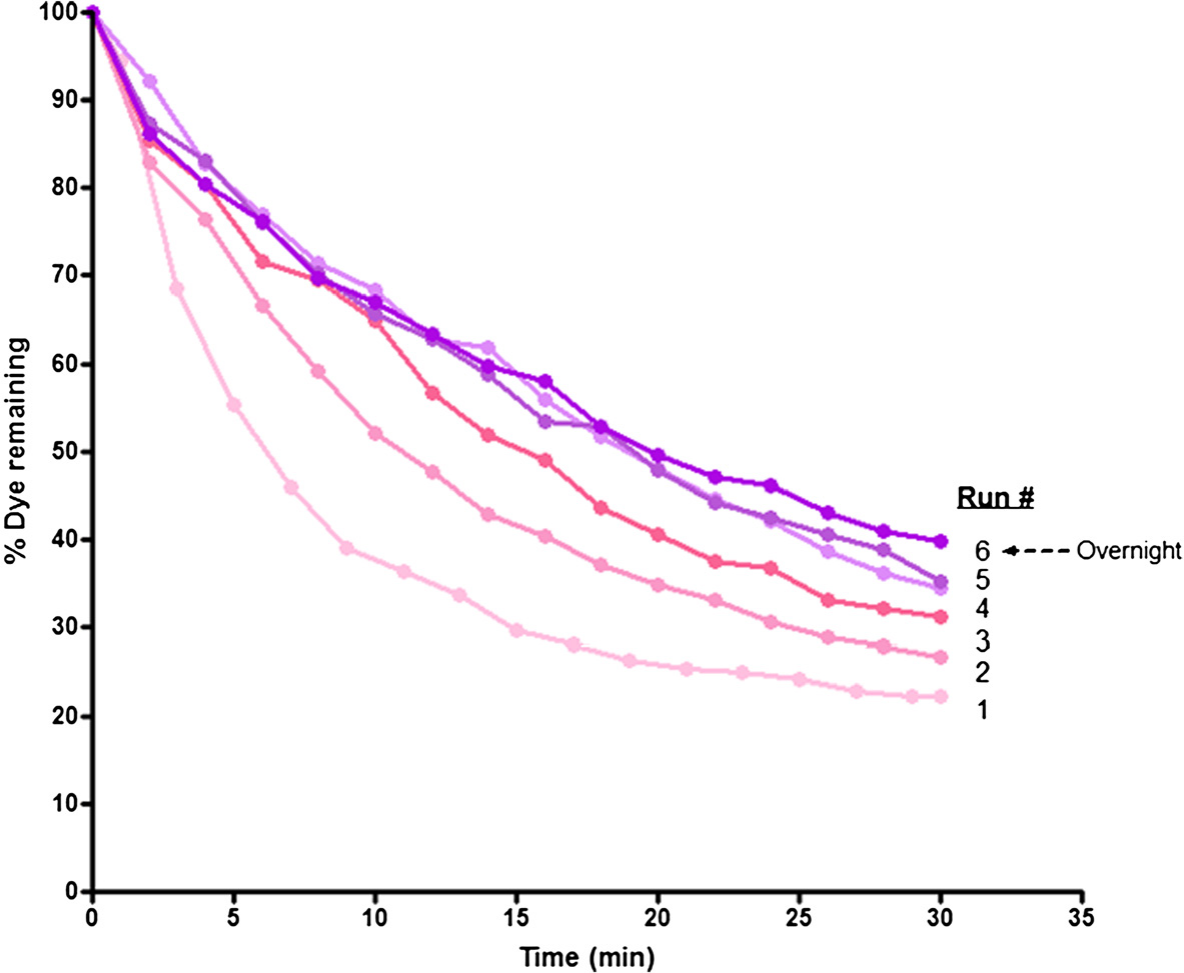
**Activity of polyacrylamide-entrapped SBP (PA-SBP) for degrading Trypan Blue.** [Dye] = 20 ppm, [H_2_O_2_] = 64 μM, pH 7. PA-SBP (approximately equivalent to 40 U/ml) was prepared as described under Materials and Methods and was added to a dye solution containing hydrogen peroxide to start the reaction. After 30 minutes, the PA-SBP was taken out and reused (up to 6 times). Run 6 was carried out using the 5-times used PA-SBP that was stored over night at 4°C in pH 7 buffer.

Immobilization of enzymes provides higher stability, reusability and capability to work in aqueous as well as in organic solvents due to protection of enzymes against denaturants, proteolysis and reduced susceptibility to microbial contamination. It is generally believed that the enzyme immobilization provide stabilization effect by restricting the protein unfolding process as a result of the introduction of random intra and intermolecular cross-links [24]. Due to these reasons, immobilization of SBP was assessed for dye degradation. In the present study, the enzyme was entrapped in a PA matrix and then used for dye degradation. Although acrylamide is toxic, the polymerized form (PA) is not and was used here to explore an additional immobilization support for enzymes. Our preliminary results showed that PA-entrapped SBP was very active and could efficiently remove about 80% of the dye in 30 minutes (Figure 6). Subsequent reuse of the same entrapped SBP was found to be still quite effective and the enzyme could be used up to six times, with only slight decrease in activity (about 60% dye degradation could be achieved in 30 minutes). This preliminary study shows the feasibility of using immobilized SBP enzyme for degradation of dyes and industrial pollutants.

### HPLC-DAD-MS/MS analysis of dye degradation

**Figure 7:**
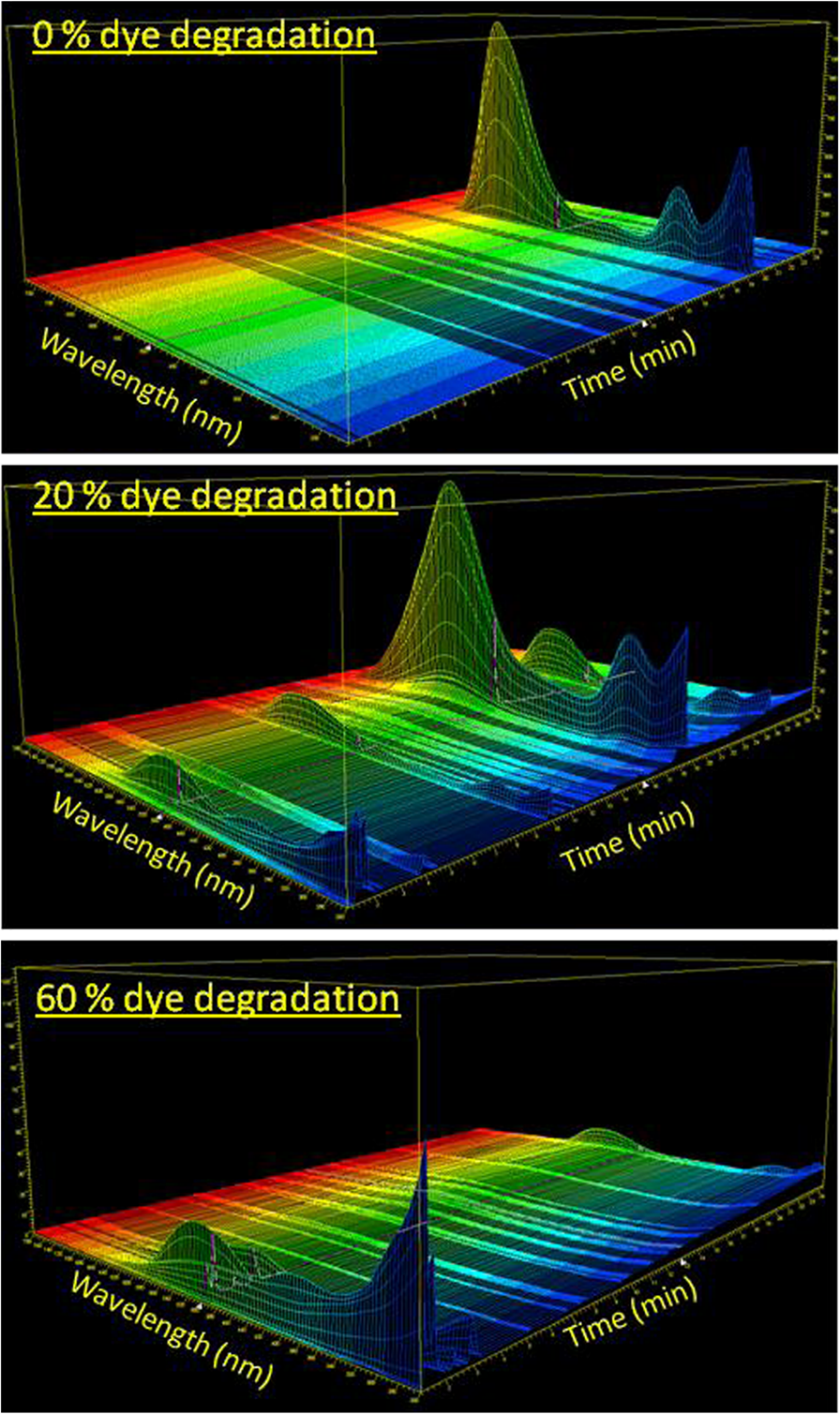
**HPLC-DAD analysis of SBP mediated Trypan Blue degradation.** HPLC analyses were carried out as described under Materials and Methods. [Dye] = 40 ppm, [H_2_O_2_] = 64 μM, [SBP] = 40 U/ml, pH 7. The sample labeled “0 % dye degradation” was pure Trypan Blue dye.

**Figure 8:**
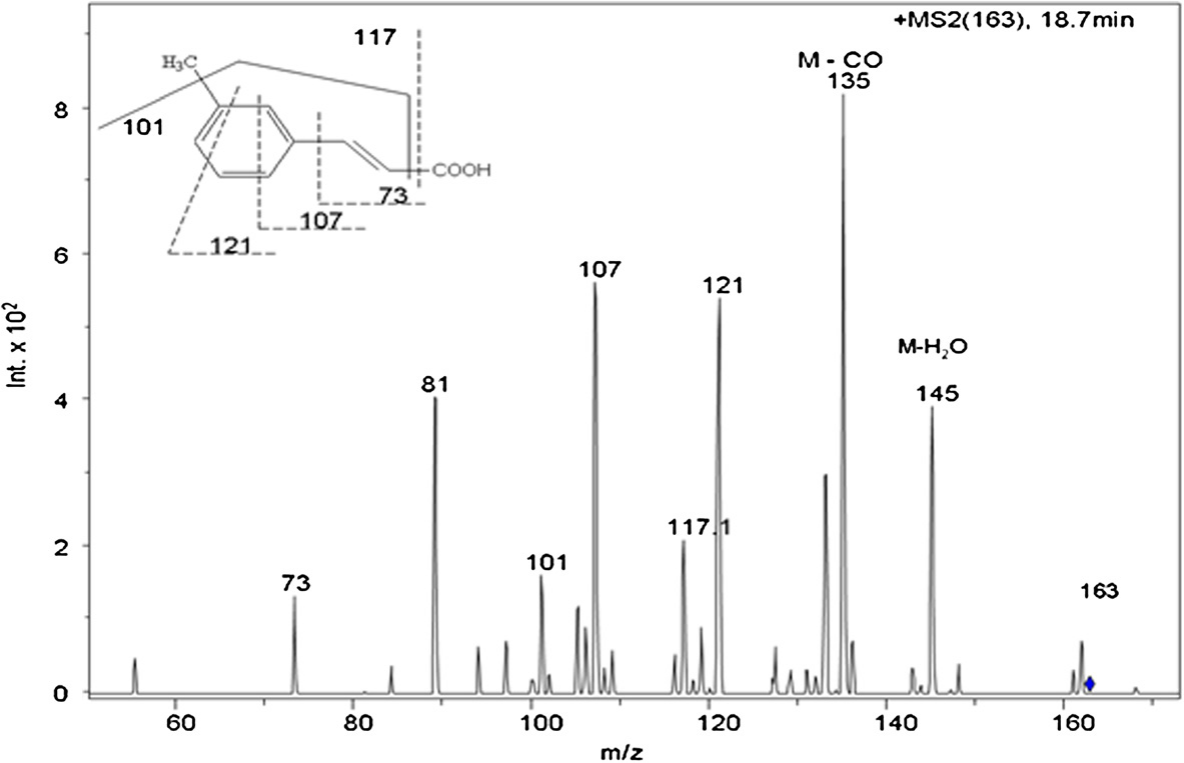
**Tandem MS/MS analysis of an intermediate with a mass of 163 Daltons (intermediate #10 as indicated in Figure**9**and Table**2**).**

**Figure 9:**
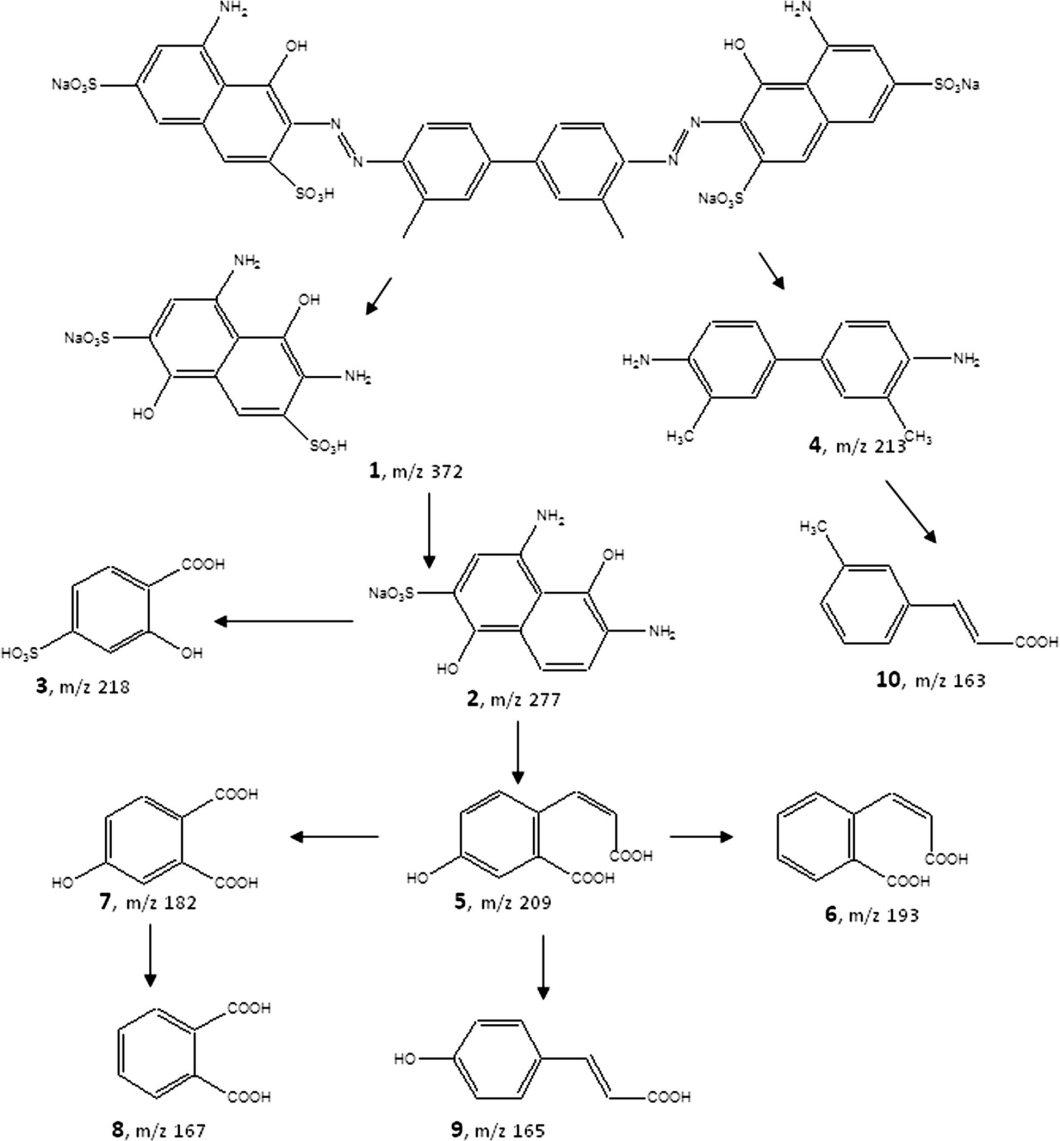
**Possible degradation mechanism of Trypan Blue based on the LC/MS/MS data (the MS/MS data for the numbered intermediates are shown in Table**2**).**

SBP-mediated degradation of Trypan Blue dye was monitored by Reverse Phase HPLC Diode Array Detector (RP-HPLC-DAD) analysis as well. Figure 7 shows the chromatograms of the pure dye, 20% degraded dye solution, and 60% degraded dye solution. As can be seen from the figure, after subjecting the dye to SBP treatment (and resulting in 20% dye degradation), the HPLC profile of the solution showed additional peaks (presumably metabolites) eluting at different retention times. This was more dramatically seen with the 60% dye degradation sample where the initially formed products were further transformed into secondary products, with totally different UV-visible spectra. In order to identify these intermediate metabolites and secondary products and to come up with a possible mechanistic pathway of SBP-mediated dye degradation, we employed LC-MS and MS/MS analyses. Using this approach, we were able to identify the molecular mass of ten different intermediates produced during SBP-H_2_O_2_-mediated degradation of Trypan Blue. The structures of these metabolites were confirmed with tandem MS-MS fragmentation analyses. Figure 8 shows a representative MS/MS fragmentation analysis of a metabolite with a mass of 163 that elutes at 18.7 min in the RP-HPLC chromatogram. Table 2 shows the molecular masses of all the ten intermediates (identified as numbers 1 through 10), as well as their fragment masses using MS-MS analyses. We have used the structures of these ten metabolites to come up with a possible scheme for the enzymatic degradation of Trypan Blue. As can be seen from Figure 9, our results show that Soybean peroxidase causes Trypan Blue degradation via symmetrical azo cleavage and subsequent radical-initiated ring opening of the metabolites. Zhang et al. have also reported such symmetrical azo dye cleavage of two different azo dyes using a slightly different chloroperoxidase [19]. Hence it appears that symmetrical azo dye cleavage may be a preferred mechanism employed by peroxidases.

**Table 2:** Summary of the tandem mass spectrometry fragment analysis of the ten Trypan Blue degradation intermediates

**Intermediate number**	**Intermediate mass**	**Fragment masses**
1	372	327, 283, 259, 227, 177, 171, 133,
2	277	251, 248, 231, 203, 179, 139
3	218	200, 173, 155, 237, 122, 111
4	213	195, 185, 167, 155
5	209	192, 167, 136, 74
6	193	175, 165, 148
7	182	137, 121, 93
8	167	149, 140, 131, 123, 109, 57,
9	165	148, 135, 121, 107,89
10	163	145, 135, 121, 117, 107, 101, 73

It is also interesting to note that some of the resulting intermediates produced during Trypan Blue degradation are aromatic amines (compounds 1, 2, and 4), however, they are quickly degraded to smaller and less-aromatic compounds (as shown in Figure 9) and may eventually be mineralized to CO_2_ and ammonium ions. We plan to carry out studies on the toxicity of intermediate solutions in the near future as well as to confirm the actual mineralization of the dye, which are slightly outside the scope of the current study. Another surprising finding of our report is that peroxidase-H_2_O_2_ mediated degradation of phenolic compounds normally produces high molecular weight polymers, which were not observed here. Perhaps the complex structure of our dye (phenolic, amines and sulphonic groups) shifted the equilibrium in favor of smaller degradation products as opposed to higher molecular weight polymers.

## Conclusion

In summary, the experimental results presented here showed the effectiveness of the peroxidase catalyzed enzymatic reaction in the degradation of Trypan Blue in aqueous phase. The performance of SBP catalyzed reaction for dye removal was found to be dependent upon the reaction time, dye concentration, enzyme concentration, H_2_O_2_ dose and pH value. The stepwise addition of H_2_O_2_ caused more than 90% degradation of the dye in less than 15 minutes. Immobilized SBP in polyacrylamide matrix also showed efficient dye degradation and could enable effective use of the same enzyme many times. HPLC-DAD analysis showed rapid formation of new products with different UV–vis spectra upon the addition of the enzyme. In addition to the above-mentioned optimization of various parameters for efficient dye degradation, our study employed extensive LC-MS and tandem MS-MS analyses to identify the intermediate metabolites produced in the process and propose a detailed mechanism of dye degradation involving symmetrical azo-bond.

## Competing interests

The authors declare that they have no competing interests.

## Authors’ contributions

UK performed the main part of the experiments. SSA supervised the experiments, helped with the experimental design, prepared the figures and helped with writing the manuscript. MAM performed the HPLC and LC-MS experiments and helped analyzing the respective data. MAR took the lead in the manuscript writing and interpreting some of the data. HNB co-supervised the work. All the authors read and approved the final manuscript.

## Supplementary Material

Additional file 1: Figure S1 Effect of dye concentration on Trypan Blue degradation. [SBP] = 40 U/ml, [H_2_O_2_] = 64 μM, pH = 7. Click here for file

Additional file 2: Figure S2 Effect of H_2_O_2_ concentration on Trypan Blue degradation. [Dye] = 10 ppm and 20 ppm, [H_2_O_2_] = 64 μM, SBP = 40 U/ml, pH = 7, degradation time = 10 minutes. Click here for file

Additional file 3: Figure S3 Effect of H_2_O_2_ concentration on Trypan Blue degradation. [Dye] = 10 ppm and 40 ppm, [H_2_O_2_] = 64 μM, SBP = 40 U/ml, pH = 7, degradation time = 10 minutes. Click here for file

Additional file 4: Figure S4 Effect of H_2_O_2_ concentration on Trypan Blue degradation. [Dye] = 10 ppm and 80 ppm, [H_2_O_2_] = 64 μM, SBP = 40 U/ml, pH = 7, degradation time = 10 minutes. Click here for file

## References

[B1] Forgas E Cserhati T Oros G Removal of synthetic dyes from wastewater: a review Env Int 2004 30 953 971 10.1016/j.envint.2004.02.001 15196844

[B2] Golka K Kopps S Myslak ZW Carcinogenicity of azo colorants: influence of solubility and bioavailability Toxicol Lett 2004 151 203 210 10.1016/j.toxlet.2003.11.016 15177655

[B3] Alam MZ Ahmad S Malik A Ahmad M Mutagenicity and genotoxicity of tannery effluents used for irrigation at Kanpur, India Ecotox Env Safety 2010 73 1620 1628 10.1016/j.ecoenv.2010.07.009 20684992

[B4] Carneiroa PA Umbuzeirob GA Oliveirac DP Zanonia MVB Assessment of water contamination caused by a mutagenic textile effluent/dyehouse effluent bearing disperse dyes J Hazard Mat 2010 174 694 699 10.1016/j.jhazmat.2009.09.106 19853375

[B5] Riera-Torres M Gutiérrez-Bouzán C Crespi M Combination of coagulation – flocculation and nanofiltration techniques for dye removal and water reuse in textile effluents Desal 2010 252 53 59 10.1016/j.desal.2009.11.002

[B6] Amini M Arami N Mahmoodi M Akbari A Dye removal from colored textile wastewater using acrylic grafted nanomembrane Desal 2011 267 107 113 10.1016/j.desal.2010.09.014

[B7] Ahmad AL Puasa SW Reactive dyes decolourization from an aqueous solution by combined coagulation/micellar-enhanced ultrafiltration process Chem Eng J 2007 132 257 265 10.1016/j.cej.2007.01.005

[B8] Alnuaimi MM Rauf MA Ashraf SS Comparative degradation study of Neutral Red by different oxidative processes Dyes Pigm 2007 72 367 371 10.1016/j.dyepig.2005.09.020

[B9] Kalsoom U Ashraf SS Meetani M Rauf MA Bhatti HN Degradation and kinetics of H_2_O_2_ assisted photochemical oxidation of Remazol Turquoise Blue Chem Eng J 2012 200–202 373 379

[B10] Rauf MA Ashraf SS Survey of recent trends in biochemically assisted degradation of dyes Chem Eng J 2012 209 520 530

[B11] Chandra R Singh R Decolourisation and detoxification of rayon grade pulp paper mill effluent by mixed bacterial culture isolated from pulp paper mill effluent polluted site Biochem Eng J 2012 61 49 58

[B12] Zucca P Rescigno A Pintus M Rinaldi AC Sanjust E Degradation of textile dyes using immobilized lignin peroxidase-like metalloporphines under mild experimental conditions Chem Cent J 2012 6 1 8 10.1186/1752-153X-6-1 23256784 PMC3567428

[B13] Jamal F Singh S Qidwai T Pandey PK Singh D Optimization of internal conditions for biocatalytic dye color removal and a comparison of redox mediator’s efficiency on partially purified Trichosanthes dioica peroxidase J Mol Catal B: Enzym 2012 74 116 124 10.1016/j.molcatb.2011.09.007

[B14] Bibi I Bhatti HN Asgher M Comparative study of natural and synthetic phenolic compounds as efficient laccase mediators for transformation of cationic dye Biochem Eng J 2011 56 225 231 10.1016/j.bej.2011.07.002

[B15] Lu L Zhao M Wang TN Zhao LY Du MH Li TL Li DB Characterization and dye decolorization ability of an alkaline resistant and organic solvents tolerant laccase from *Bacillus licheniformis* LS04 Biores Tech 2012 115 35 40 10.1016/j.biortech.2011.07.111 21868217

[B16] Saladino R Guazzaroni M Crestini C Crucianelli M Dye Degradation by Layer‒by‒Layer Immobilised Peroxidase/Redox Mediator Systems Chem Cat Chem 2013 10.1002/cctc.201200660

[B17] Meetani MA Hisaindee SM Abdullah F Ashraf SS Rauf MA Liquid chromatography tandem mass spectrometry analysis of photodegradation of a diazo compound: a mechanistic study Chemosphere 2010 80 422 427 10.1016/j.chemosphere.2010.04.065 20529695

[B18] Satar R Husain Q Applications of Celite-adsorbed white radish (*Raphanus sativus*) peroxidase in batch process and continuous reactor for the degradation of reactive dyes Biochem Eng J 2009 46 96 104 10.1016/j.bej.2009.04.012

[B19] Zhang J Feng M Jiang Y Hu M Li S Zhai Q Efficient decolorization/ degradation of aqueous azo dyes using buffered H_2_O_2_ oxidation catalyzed by a dosage below ppm level of chloroperoxidase Chem Eng J 2012 191 236 242

[B20] Laurenti E Ghibaudi E Ardissone S Ferrari RP Oxidation of 2,4-dichlorophenol catalyzed by horseradish peroxidase: characterization of the reaction mechanism by UV–visible spectroscopy and mass spectrometry J Inorg Biochem 2003 95 171 176 10.1016/S0162-0134(03)00101-6 12763662

[B21] Marchis T Avetta P Prevot AB Fabbri D Viscardi G Laurenti E Oxidative degradation of Remazol Turquoise Blue G 133 by soybean peroxidase J Inorg Biochem 2011 105 321 327 10.1016/j.jinorgbio.2010.11.009 21194634

[B22] McEldoon JP Dordick JS Unusual thermal stability of soybean peroxidase Biotechnol Progress 1996 12 555 558 10.1021/bp960010x

[B23] Kamal JKA Behere DV Kinetic stabilities of soybean and horseradish peroxidases Biochem Eng J 2008 38 110 114 10.1016/j.bej.2007.07.019

[B24] Rogalski J Jozwik E Hatakka A Leonomicz A Immobilization of laccase from Phlebia radiata on controlled porosity glass J Mol Catal B: Enzym 1995 95 99 108 10.1016/1381-1169(94)00165-0

