# Supplementary figures and images for: Mechanistic study of a diazo dye degradation by Soybean Peroxidase
Source: Chem Cent J. 2013 May 27;7:93. doi: 10.1186/1752-153X-7-93 (PMC3680093; doi:10.1186/1752-153X-7-93)

**Additional file 1: Figure S1**


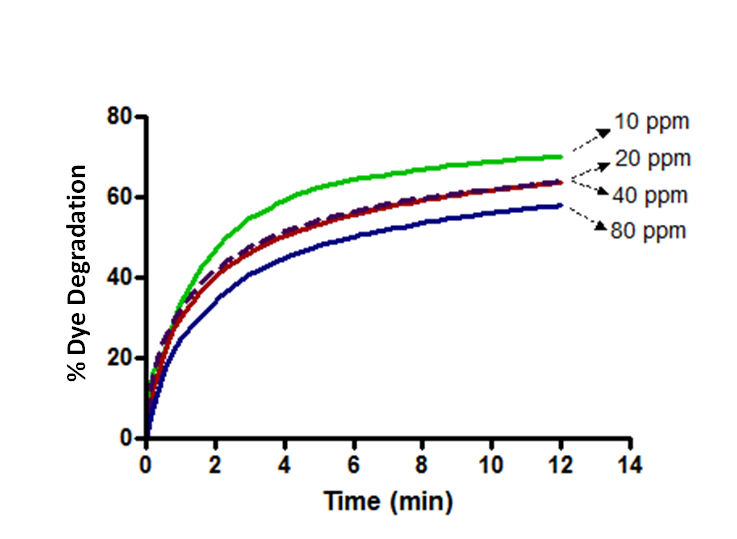

Supplement: Additional file 1: Figure S1 — Effect of dye concentration on Trypan Blue degradation. [SBP] = 40 U/ml, [H2O2] = 64 μM, pH = 7. [file 1752-153X-7-93-S1.doc]

**Additional file 2: Figure S2**

**
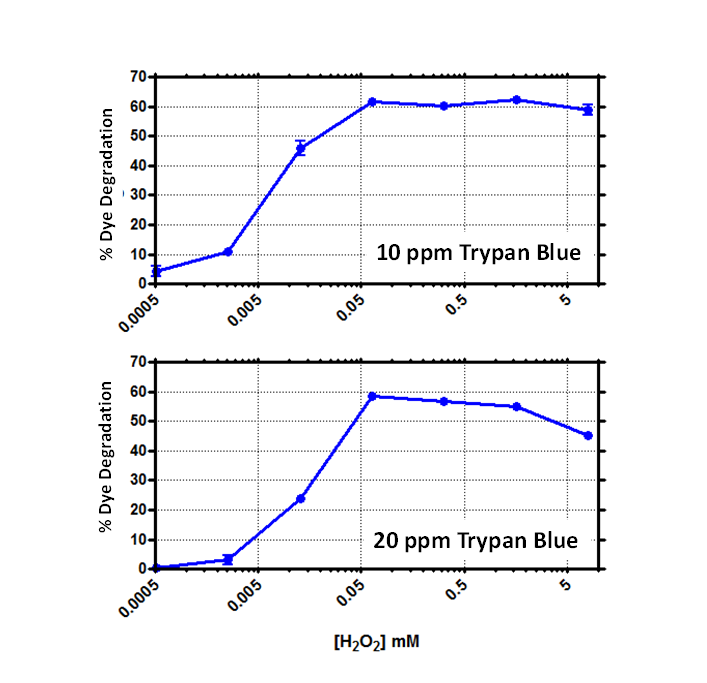
**

Supplement: Additional file 2: Figure S2 — Effect of H2O2 concentration on Trypan Blue degradation. [Dye] = 10 ppm and 20 ppm, [H2O2] = 64 μM, SBP = 40 U/ml, pH = 7, degradation time = 10 minutes. [file 1752-153X-7-93-S2.doc]

**Additional file 3: Figure S3**

**
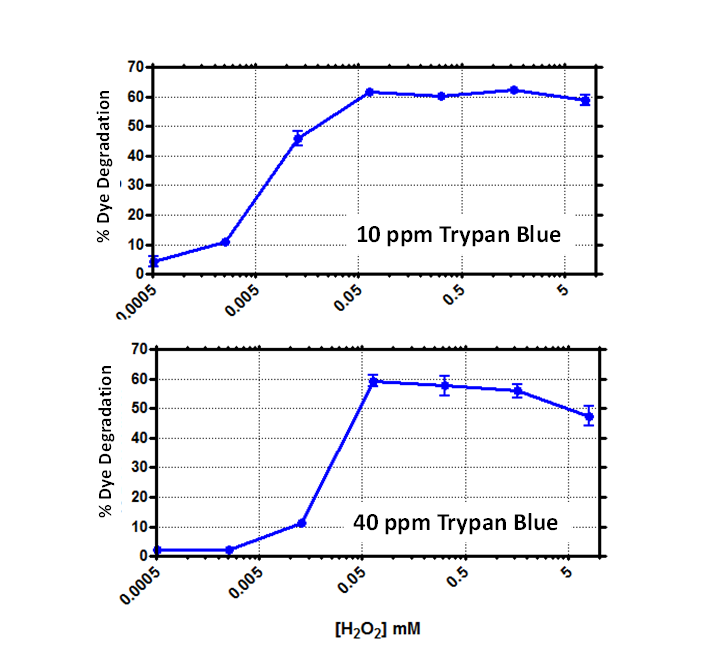
**

Supplement: Additional file 3: Figure S3 — Effect of H2O2 concentration on Trypan Blue degradation. [Dye] = 10 ppm and 40 ppm, [H2O2] = 64 μM, SBP = 40 U/ml, pH = 7, degradation time = 10 minutes. [file 1752-153X-7-93-S3.doc]

**Additional file 4: Figure S4**

**
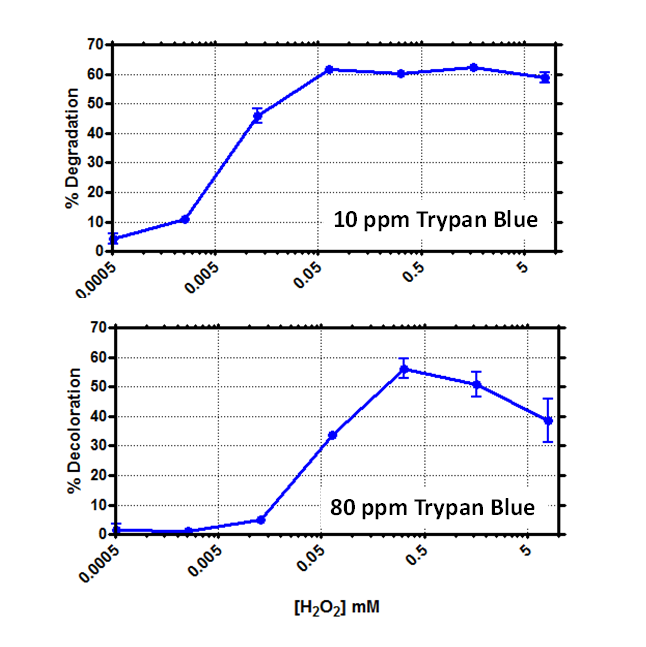
**

Supplement: Additional file 4: Figure S4 — Effect of H2O2 concentration on Trypan Blue degradation. [Dye] = 10 ppm and 80 ppm, [H2O2] = 64 μM, SBP = 40 U/ml, pH = 7, degradation time = 10 minutes. [file 1752-153X-7-93-S4.doc]
